# SMARTERscreen protocol: a three-arm cluster randomised controlled trial of patient SMS messaging in general practice to increase participation in the Australian National Bowel Cancer Screening Program

**DOI:** 10.1186/s13063-023-07756-5

**Published:** 2023-11-13

**Authors:** Jennifer G. McIntosh, Jon D. Emery, Anna Wood, Patty Chondros, Belinda C. Goodwin, Judy Trevena, Carlene Wilson, Shanton Chang, Jane Hocking, Tina Campbell, Finlay Macrae, Kristi Milley, Jie-Bin Lew, Claire Nightingale, Ian Dixon, Makala Castelli, Nicholas Lee, Lyle Innes, Tamara Jolley, Sabine Fletcher, Lyn Buchanan, Sally Doncovio, Kate Broun, Glenn Austin, Joyce Jiang, Mark A. Jenkins

**Affiliations:** 1 Centre for Cancer Research, University of Melbourne, Parkville, Australia; 2 Department of General Practice and Primary Care, University of Melbourne, Parkville, Australia; 3 Centre for Epidemiology and Biostatistics, University of Melbourne Centre for Epidemiology and Biostatistics, Melbourne School of Population and Global Health, University of Melbourne, Parkville, Australia; 4 Cancer Council Queensland, Fortitude Valley, QLD Australia; 5 Centre for Health Research, University of Southern Queensland, Springfield, Australia; 6 School of Computing and Information Systems, The University of Melbourne, Parkville, Australia; 7 Healthily Pty Ltd, Melbourne, Australia; 8 Colorectal Medicine and Genetics, The Royal Melbourne Hospital, Parkville, Australia; 9 Department of Medicine, University of Melbourne, Melbourne, Australia; 10 Primary Care Collaborative Cancer Clinical Trials Group (PC4), Melbourne, Australia; 11 The Daffodil Centre, a joint venture between Cancer Council NSW and the University of Sydney, Sydney, Australia; 12 Consumer Representative, Melbourne, Australia; 13 Victorian Department of Health, Melbourne, Australia; 14 Cancer Council Victoria, Melbourne, Australia; 15 Queensland Health, Brisbane, Australia; 16 Multicultural Centre for Women’s Health, Melbourne, Australia

**Keywords:** Colorectal cancer screening, Australian National Bowel Cancer Screening Program, General practice, Behaviour change

## Abstract

**Background:**

Australia persistently has one of the highest rates of colorectal cancer (CRC) in the world. Australia’s National Bowel Cancer Screening Program (NBCSP) sends a biennial Faecal Immunochemical Test (FIT)—the ‘NBCSP kit’—to everyone eligible for the programme between 50 and 74 years old; however, participation in the programme is low, especially in the 50- to 60-year-old age group. Our previous efficacy trial (‘SMARTscreen’) demonstrated an absolute increase in uptake of 16.5% (95% confidence interval = 2.02–30.9%) for people sent an SMS with motivational and instructional videos, from their general practice prior to receiving their NBCSP kit, compared to those receiving usual care. Building on the strengths of the SMARTscreen trial and addressing limitations, the ‘SMARTERscreen’ trial will test the effect on participation in the NBCSP of sending either an SMS only or an SMS with online video material to general practice patients due to receive their NBCSP compared to ‘usual care’.

**Methods:**

SMARTERscreen is a three-arm stratified cluster randomised controlled trial involving 63 general practices in two states in Australia. Eligible patients are patients who are aged 49–60 years and due to receive their NBCSP kit within the next 2 weeks during the intervention period. General practices will be equally randomised to three trial arms (21:21:21, estimated average 260 patients/practice). The two interventions include (i) an SMS with an encouraging message from their general practice or (ii) the same SMS with weblinks to additional motivational and instructional videos. The control arm will receive ‘usual care’. Using the intention-to-treat approach, primary analysis will estimate the three pair-wise between-arm differences in the proportion of eligible patients who participate in the NBCSP within 6 months of when their kit is sent, utilising screening data from the Australian National Cancer Screening Register (NCSR). Patient intervention adherence to the interventions will also be evaluated. Findings will be incorporated into the Policy1-Bowel microsimulation model to estimate the long-term health benefits and cost-effectiveness of the interventions.

**Discussion:**

SMARTERscreen will provide high-level evidence determining whether an SMS or an SMS with web-based material sent to general practice patients prior to receiving their NBCSP kit increases participation in bowel cancer screening.

**Trial registration:**

Australian New Zealand Clinical Trials Registry ACTRN12623000036617. Registered on 13 January 2023.

Trial URL: https://www.anzctr.org.au/Trial/Registration/TrialReview.aspx?id=385119&isClinicalTrial=False

## Introduction

### Background and rationale {6a}

Australia has one of the highest rates of colorectal cancer (CRC) in the world [1]. Currently, 40% of CRC cases are diagnosed at stage 3 or 4 leading to a poorer prognosis [2]. Screening for precancerous adenomas and early-stage cancer, at which time they can be easily treated, improves outcomes and is cost-effective [3, 4]. Australia has a National Bowel Cancer Screening Program (NBCSP), which is a coordinated, population-based screening programme that sends immunochemical Faecal Occult Blood Test (FIT) kits to eligible Australians aged 50 to 74 years every 2 years. The kits are free and sent directly to a person’s home where two samples can be self-collected and returned for testing [5]. Despite the convenience of this process, uptake of the NBCSP is only 40.9% with fewer people in the younger age groups completing the kit; currently, only 31.6% of people between 50 and 54 years return the kit for testing [6]. Modelling has estimated that if screening participation increased by an additional 10%, 24,300 additional CRC diagnoses and 16,800 additional CRC deaths could be prevented, and an additional $300 million dollars in healthcare expenditure saved over the next 25 years in the Australian population [7].

Multiple strategies have been tested to increase CRC screening uptake, all having varying degrees of success either as single or multifaceted interventions [8]. Of these, endorsement from a patient’s general practitioner (GP) has been demonstrated to have one of the biggest impacts on increasing uptake [9, 10]. Direct engagement by GPs with their patients increases patient awareness about screening and reduces anxiety and fear about participating in screening [11]. In Australia, the kit is sent directly to the participant from the NBCSP, and currently, there is no coordinated and efficient way for the GP to prompt or encourage their patients to participate in screening.

Short message services (SMS) are being used more often by general practice to communicate with patients because this approach provides an opportunity to reach large numbers of patients in real time and messages can be viewed discreetly multiple times at an individual’s convenience and have demonstrated success at increasing screening uptake internationally[12, 13]. Between 2020 and 2021, we undertook a trial in 21 general practices, called SMARTscreen to test an intervention which combined, in one SMS, multiple evidence-based components known to increase screening uptake including a message of endorsement from a credible source (i.e. the GP) and weblinks to motivational video narratives and instructions for how to do the test [14]. The SMS was sent to patients from their general practice just before they were due to receive their NBCSP kit. The SMARTscreen trial demonstrated that sending SMS prompts from a patient’s general practice increased NBCSP kit return by 16.5% (95% confidence interval = 2.0–30.9%; 39% kit return in the intervention practice compared with 23% in control practices) [15] and was acceptable and feasible to both practice staff and patients (in submission). Generalisability of the results was limited because only one regional location in Australia was involved and, as the data were collected at the aggregated practice level, this meant that the analysis by individual patient characteristics was limited. We were also unable to differentiate between the effect of receiving the SMS and the contribution of the materials accessed via weblink within the SMS message (i.e. video content) [15].

Another limitation was that the date the patient’s NBCSP kit was due in the SMARTscreen trial was approximated from either birthdate or previous kit return date data recorded in general practice electronic health record (EHR). Recently, the Australian Government launched a National Cancer Screening Register (NCSR) that enables a coordinated approach to invitations, reminders, and follow-up for bowel and cervical cancer screening. The NCSR allows GPs to directly access their patients’ screening status, including when the next kit is due and their screening history through their electronic medical software [16]. Using the NCSR data will provide a more accurate date for the FIT arrival to inform the timing of SMS interventions.

The SMARTERscreen trial builds on the strengths and addresses the limitations of SMARTscreen. This trial will involve testing the effect of an SMS alone or the SMS in combination with a web-based link with revised video content, compared with usual care in a larger and more diverse general practice population across metropolitan and rural areas. The due date for when the NBCSP kit will be sent, and the outcome of screening participation will be collected directly from the NCSR records instead of relying on general practice electronic health records and therefore will provide more reliable information. Individual patient characteristics will be collected from the NCSR and practices providing more information about the impact of the interventions on screening behaviour by age, sex, previous screening history, and location (based on the geographical location of the patients’ general practice).

## Objectives {7}

SMARTERscreen is a three-arm parallel cluster randomised controlled superiority trial in general practices in the Australian states of Victoria and Queensland. The general practices will be allocated on 1:1:1 ratio to test all three pair-wise comparisons between arms:

1. Control arm: practices continue with usual care in which general practitioners continue with opportunistic discussions about bowel cancer screening;
2. Intervention arm 1: an ‘SMS only’ message sent to the patient from their general practice advising them that their FIT kit will be coming in the mail soon and that their GP strongly advises that they complete it
3. Intervention arm 2: an ‘SMS bundle’ is sent which is the same message as for intervention 1, but with a weblink to extra online information and resources designed to increase participation in the NBCSP.

The trial aims to assess whether sending either an SMS alone or an SMS in combination with a web-based link to additional motivational resources to 49- to 60-year-old general practice patients who are due to receive their kit from the NBCSP will increase CRC screening uptake in the programme within 6 months of when their kit is due compared to the control arm, respectively. Further, the trial aims to assess whether including a web-based link in the SMS to motivational and instructional videos increases screening uptake compared to SMS alone. Patients’ screening status, defined as having a recorded FIT result within 6 months of when their kit is due, will be extracted at the individual level from the NCSR.

Secondary aims will be:

- To identify patient characteristics, including age, sex, previous screening, and location of practice, that modify the intervention effect of SMS only and SMS bundle compared to the control on proportion who uptake CRC screening within 6 months
- To evaluate adherence to the intervention by measuring the number of SMS/SMS bundles unable to be delivered to patients relative to the number sent, the proportion of people who opt out of receiving more SMS/SMS bundles, the proportion of people who receive the SMS bundle who open the SMS weblink and view the videos
- To evaluate the cost-effectiveness of the two interventions compared to usual care and potential health cost savings if a SMS intervention were to be adopted and implemented nationally. This objective includes estimating the potential number of lives saved by increasing screening uptake.

### Hypotheses

Our primary hypotheses are:

1. A GP practice-endorsed SMS sent from general practice to patients aged between 49 and 60 years old and due for a NBCSP kit will increase the proportion of patients who return the NBCSP kit within 6 months of when their kit is sent compared to usual care;
2. A SMS bundle with a GP endorsement of the NBCSP and additional material (i.e. motivational and instructional videos) from general practice to patients aged between 49 and 60 years old and due for a NBCSP kit will increase the proportion of patients who return the NBCSP kit within 6 months of when their kit is sent compared to usual care;
3. Proportion of general practice patients who return the NBCSP kit within 6 months of when the kit was due will differ between patients aged between 49 and 60 years old and due for a NBCSP kit who receive SMS bundle with a GP endorsement of the NBCSP and additional material (i.e. motivational and instructional videos) compared to those who receive an SMS with only GP endorsement of the NBCSP.

Secondary hypotheses:

Sending a GP practice-endorsed SMS with/without additional motivational material to people before their kit is sent will be cost-effective compared with usual care.

## Trial design {8}

SMARTERscreen is a stratified cluster randomised controlled superiority trial in 63 general practices randomised equally into one of three arms (21:21:21), using block randomisation within four strata [Victoria vs Queensland (50% from each State), and metropolitan/larger regional vs rural/smaller regional location (60% from metropolitan/larger rural, 40% rural/smaller regional locations) of the general practice].

## Methods: participants, interventions, and outcomes

### Study setting {9}

General practices in Queensland and Victoria, Australia.

### Eligibility criteria {10}

#### Inclusion and exclusion criteria for general practices

Practices will be included if they are in Queensland or Victoria, use electronic health record (EHR) software compatible with the National Cancer Screening Register (NCSR) (Best Practice, Medical Director Version 4) and are willing to download the free NCSR application which provides a portal between the NCSR and the general practice EHR. Practices will be eligible if they have at least two full-time equivalent (FTE) GPs working in their general practice and have a practice manager (or delegate) who will champion the study throughout the trial period. General practices geographically located in very remote areas, as defined by the Modified Monash Model (MMM) category 7 [17], which includes offshore and central Australian locations, will be excluded for logistical reasons [17].

Practices will not be approached where it is known that they have been involved in recent research projects in cancer screening or are involved in other bowel cancer research projects at the University of Melbourne, or cancer screening quality improvement programmes for example those conducted by the local Primary Health Networks.

Under the NBCSP ‘hot zone policy’, the NBCSP suspends sending out kits for up to 6 months of the year to certain areas defined by postcode due to extreme heat during summer (correspondence from the NBCSP). Practices will be ineligible if they are in areas classified as ‘hot zones’ as their patients will not receive a kit during some or all the trial intervention period. ‘Hot zones’ account for 111 (25%) of 447 postcodes and 367 (23%) of practices in Queensland (none in Victoria), and therefore practices sampled will still represent most practices in Queensland [5]. General practices in Australia can operate as independent small businesses or as larger businesses with multiple different clinics. When practices in different physical locations have combined EHRs for patients at all practices, these practices will be treated as one practice in the trial. The “main practice” will be defined as the practice location that the owner or staff identify as being the principal practice. If the practice is in the treatment group, the phone number (and logo) of the main practice will be the one sent in the SMS to all patients.

When more than one practice shares EHRs but identify as separate practices (i.e. they do not share the same logo and/or name), and whose patient records cannot be separated, they will not be included in the trial. In the unusual case where more than one practice shares EHRs, but if one is in a hot zone and one is not, only the patients living outside of the hot zone will be included in the study. This will be defined by patient residence postcode in the EHR.

#### Inclusion and exclusion criteria for patients

Eligibility:

- 1) Assessed at the general practice.
  People will be eligible if:
  - They are aged between 49 and 60 years old during the trial period.
  - They are a regular patient at a general practice recruited into the trial (defined by their patient file having been opened at least three times in the previous 2 years).
  - They have a mobile phone number recorded in the practice.
  - They have a Medicare number recorded in the practice.
  - They have not opted out of receiving SMS from their practice.
  - They do not have a diagnosis of CRC in their EHR.
- 2) Assessed at the NCSR.
  People identified as eligible in the general practice records will be linked with NCSR records and remain eligible for the trial if:
  - They have matching record in the NCSR database.
  - They are due to receive their NBCSP kit within the trial period.
    People will be excluded if:
  - Their record extracted from the general practice EHR does not match with the records in the NCSR database.
  - They have a diagnosis of CRC recorded in their NCSR record.
  - They have opted out from receiving the NBCSP kit, as recorded in their NCSR record.
  - They have put their NBCSP kit on hold, as recorded in their NCSR record.
  - They have died, as recorded in their NCSR record.
  - They are not due for screening because they have had a recent colonoscopy, as recorded in their NCSR record.
  - They are not due for screening because they have had a recent FIT elsewhere, as recorded in their NCSR record.

### Who will take informed consent? {26a}

Recruitment will be overseen by the SMARTERscreen steering group (JM, MJ, BG, JE, PC, and JT) who will report to the investigators.

#### General practice informed consent for the trial

The project officers will obtain informed consent from all eligible and interested general practices. All GPs in the practice need to agree to be involved, but only one consent form will be required from each practice. Two senior practice staff—usually the Practice Manager and Principal GP or their delegate—will complete the consent form on behalf of the general practice. This is common practice in general practice research.

The practice will be provided with copies of the plain language statement and a signed consent form for their records.

#### Patient-informed consent for the trial

Patient consent is not being sought because only de-identified data will be collected for the analysis and only aggregated results will be published. Patients provide consent for the use of their health information when they join a practice, and this includes data from sites that provide access to the Provider Digital Access (PRODA) portal, an online identity verification and authentication system that lets GPs securely access government online services including the NCSR [16]. Recently, the NCSR have built a portal (the Health Provider Portal—‘HPP’) so the data transfer between practice and the NCSR can occur in real time, for example during a consultation. Using the HPP, the GP can check if a patient is due for screening. To avoid having to do this for every eligible individual (potentially 100 s of patients per practice), we have developed a way for the practice to do this in bulk using a secure file transfer portal (SFTP). To ensure the secure transfer of data between the practice and the NCSR has been established, we require the practice to have the NCSR HPP installed as part of their involvement in the trial. The transfer details are described below.

### Additional consent provisions for collection and use of participant data and biological specimens {26b}

Not applicable. No biological specimens will be collected.

### Interventions

#### Explanation for the choice of comparators {6b}

Control arm practices: GPs will continue practising usual care, complying with bowel cancer screening guidelines as defined by the Royal Australian College of General Practitioners Red Book for Preventive Activities in General Practice and opportunistically discussing bowel cancer screening with their patients [18].

#### Intervention description {11a}

The trial is comparing two interventions.

**Fig. 1:**
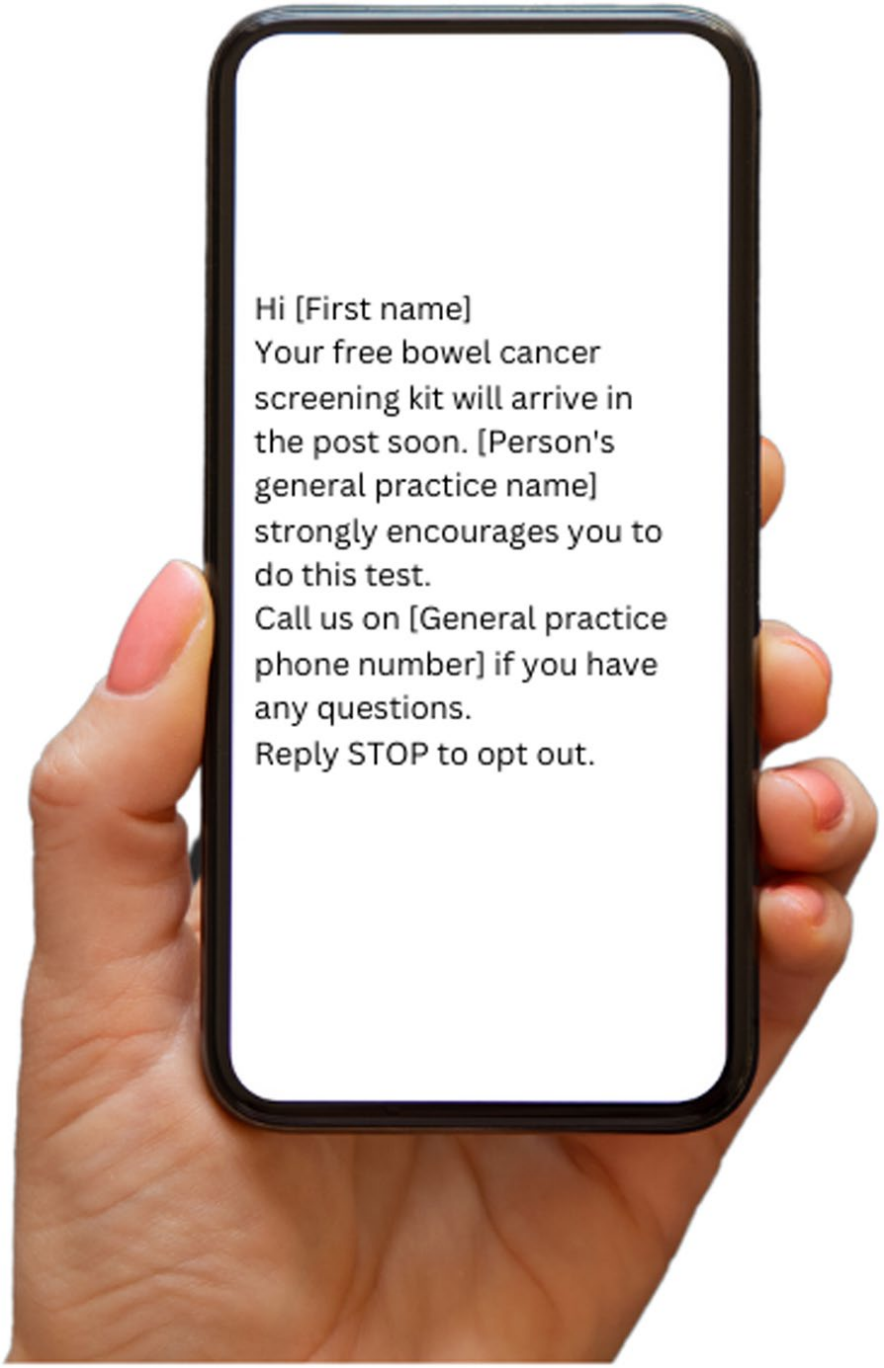
Intervention 1, the SMS only

*Intervention 1*: ‘SMS only’—an SMS will be sent from the general practice to prompt patients to do the NBCSP kit. The SMS contains a personalised greeting to the patient using their first name only, the general practice name and telephone number, and a GP endorsement of the NBCSP (Fig. 1). The SMS will be delivered by GoShare, an online tool developed by Healthily, a company that sends timely educational resources to consumers directly from their general practice via SMS [19]. Within the SMS, participants will be provided the opportunity to opt out of receiving any further health promotion SMS from Healthily, but this will not stop them from receiving other SMS messages from their practice (e.g. appointment reminders). The SMS wording is: ‘Hi [insert first name of patient here], Your free bowel cancer screening kit will arrive in the post soon. [insert general practice name here] strongly encourages you to do this test. Call us on [insert general practice phone number here] if you have any questions. Reply STOP to opt out.’ 3.

**Fig. 2:**
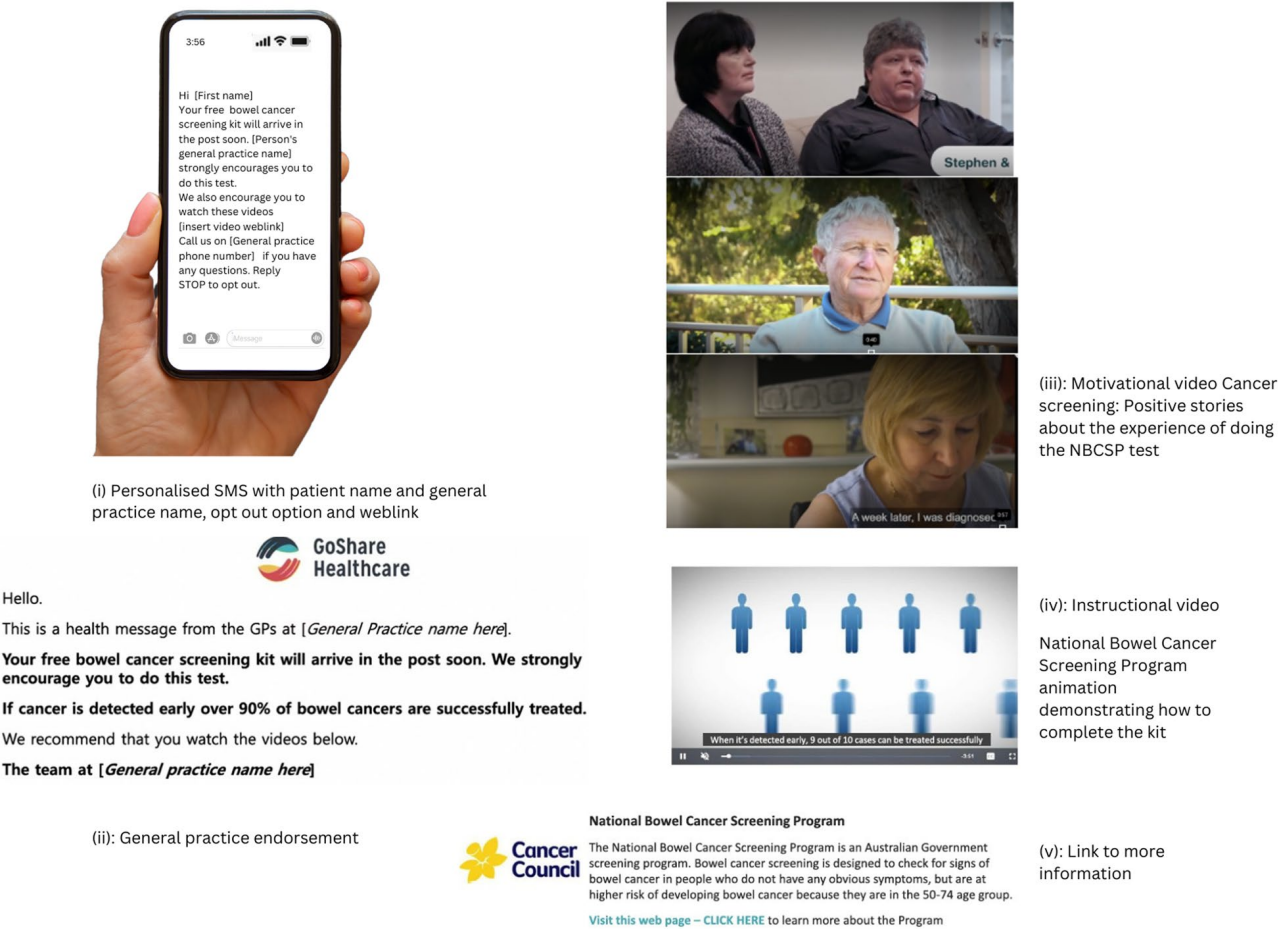
Intervention 2, the SMS bundle with the SMS with a weblink (i) and contents (ii–iv)

*Intervention 2*: ‘SMS bundle’—an SMS with a weblink will be sent from the general practice to prompt patients to do the NBCSP kit (Fig. 2). The SMS will consist of the same text message as Intervention 1 but with an added weblink to the following motivational and instructional materials: a GP endorsement of the NBCSP, a consumer co-designed video of relatable people talking about why it is important to participate in the NBCSP, an animated instructional video to provide simple step-by-step instructions on how to complete the NBCSP kit, and a link to more information about the NBCSP. The wording is: ‘Hi [insert first name of patient here], Your free bowel cancer screening kit will arrive in the post soon. [insert general practice name here] strongly encourages you to do this test. We also recommend you watch these videos [weblink to videos inserted here]. Call us on [insert general practice phone number here] if you have any questions. Reply STOP to opt out.’ The first part of the weblink shows a similar message from the general practice with the GP logo. The second and third parts include video material co-designed by Cancer Council Queensland, tested with 200 consumers and a group of experts, led by BG and the SMARTERscreen steering group. The motivational video (second part of the bundle) is a montage of three people (real consumers) discussing the benefits of doing the NBCSP kit. The instructional video (third part of the bundle) is an edited version from the NBCSP and demonstrates how to do the test (unpublished). There is also a link to more information about the NBCSP.

#### Criteria for discontinuing or modifying allocated interventions {11b}

General practices can withdraw from the SMARTERscreen trial at any time without providing a reason, but after the practices are randomised, we will not be able to exclude their patient data as they are collected in a de-identified form and will be included as part of the main analyses even if they receive part or none of the intended intervention. Patients can opt out of receiving any more SMS from Healthily by replying “STOP” to the text message. This will not stop them from receiving messages from their GP, only block future messages from Healthily. Posters will be in the waiting room of all practices to inform patients about the trial and to let them know they can ask not to be included in the trial; this will only be possible prior to de-identified data collection from the practice EHR.

Patients who are eligible to receive a NBCSP kit at the beginning of the intervention period after randomisation occurred but are subsequently not sent a FIT kit during the trial intervention period (coded as “FIT kit not sent’) will be excluded from the primary analysis. Reasons patients are not sent a kit during the 6-month intervention period may be as follows: (a) they opted out of receiving kits from the NBCSP; (b) they put their NBCSP kits on temporary hold; (c) they have a recently recorded bowel cancer diagnosis; (d) they have recently had a colonoscopy (and are not due screening); or (e) they have a record of having had a recent FIT test elsewhere.

#### Strategies to improve adherence to interventions {11c}

The SMARTERscreen project officers will be in regular contact with general practice staff during the data collection and transfer, and to manage and schedule SMS to be sent through the GoShare platform for intervention practices. Training and a comprehensive manual will be provided to maintain consistency and quality of the intervention delivery and data collection for all participating general practices.

#### Relevant concomitant care permitted or prohibited during the trial {11d}

There is no concomitant care that will be prohibited during the trial.

#### Provisions for post-trial care {30}

At the conclusion of the trial, the Healthily GoShare messaging platform will be provided free of charge to all participating general practices for 6 months. The SMARTERscreen SMS and SMS bundle with a training manual and ‘cheat sheets’ will be available ongoing. SMS messages will be subsidised by the project for 6 months for up to 260 eligible patients.

No additional post-trial care will be required as all practices will be working within the recommended clinical guidelines for CRC screening during the trial period [18].

#### Outcomes {12}

The primary outcome is the difference in all three pair-wise comparisons between the three arms in the proportion of eligible patients who were sent a NBCSP kit and who have a date recorded for when the FIT kit was received by the NBCSP and recorded in the NCSR (indicating they have returned their kit) within 6 months from the date when the kit was due to be sent to each participant. For patients who were sent the NBCSP kit, the outcome variable will be coded as having either a ‘FIT kit returned’ or ‘FIT kit not returned’. ‘FIT kit returned’ will include patients who have a date for the returned NBCSP kit in the NCSR registry within 6 months of when their kit was due. ‘FIT kit not returned’ will include patients who were sent a kit but they either do not have a date recorded, or the date is outside the 6-month range. After randomisation has occurred, patients who were eligible to receive a NBCSP kit at the beginning of the intervention period but were subsequently not sent a FIT kit during the trial intervention period will be coded as ‘FIT kit not sent’. This will include patients who were not sent a kit during the 6-month intervention period for a number of reasons: (a) they opted out of receiving kits from the NBCSP; (b) they put their NBCSP kits on temporary hold; (c) they have a recently recorded bowel cancer diagnosis; (d) they have recently had a colonoscopy (and are not due screening); or (e) they have a record of having had a recent FIT test elsewhere. These people will be considered ineligible.

#### Economic evaluation outcome

The economic model-estimated cost-effectiveness of both sending a GP practice-endorsed SMS with/without additional motivational material to people before they are due to do their NBCSP kit to increase the uptake of the NBCSP and the SMS intervention compared with usual care.

#### Measures for adherence to intervention

1. Proportion of individuals sent an SMS and was delivered in Intervention 1
2. Proportion of individuals sent an SMS bundle and was delivered in Intervention 2
3. For Intervention 2 only; proportion of individuals who receive the SMS bundle, who:
  - Open the SMS link one or more times
  - View the motivational video one or more times
  - View the instructional video one or more times
  - View the NBCSP webpage information one or more times
4. For Intervention 2 only; of individuals who open the SMS bundle, count of the:
  - Number of times the SMS link is opened
  - Number of times the instructional video is viewed
  - Number of times the motivational video is viewed
  - Number of times the NBCSP webpage information is viewed.
5. Number of people who opt out

#### Participant timeline {13}

See Fig. 3.

**Fig. 3:**
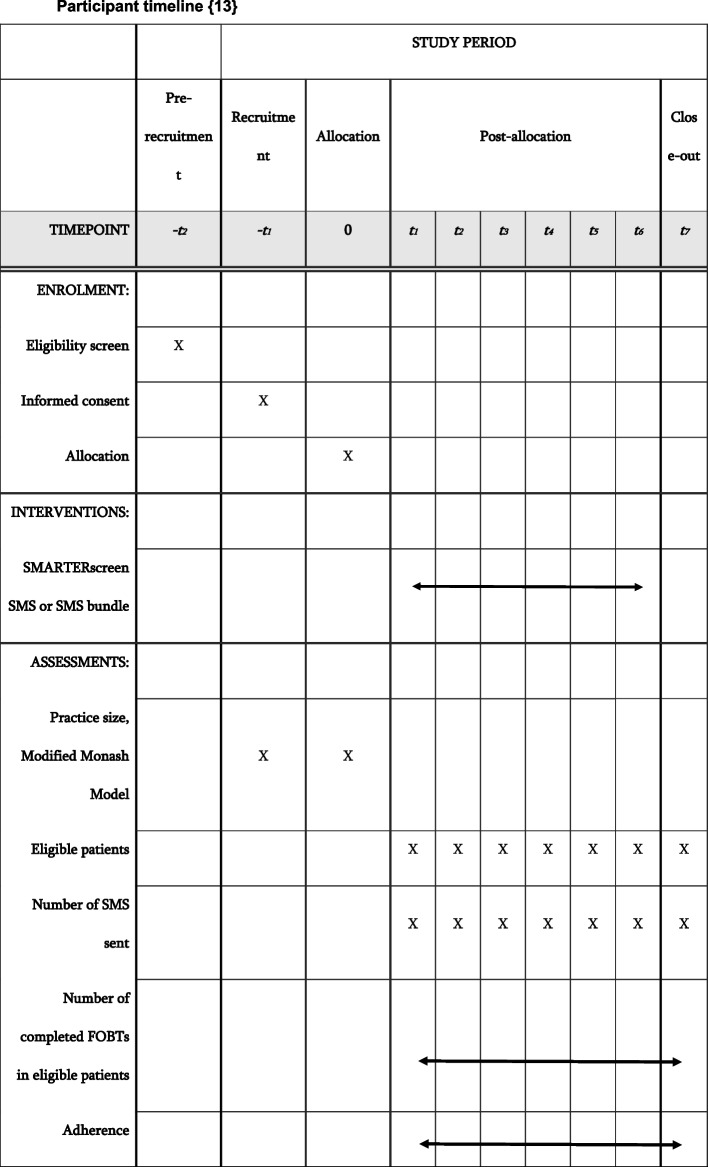
The timeline for recruitment and data collection

#### Sample size {14}

Sample size was based on 80% power for an overall two-sided significance level of 5% (alpha), and an intraclass correlation coefficient (ICC) of 0.01. Planned primary comparisons are the two intervention arms (SMS only and SMS bundle) with the control arm, respectively, and the SMS only intervention with the SMS bundle. The Holm-Bonferroni correction was used to control the family-wise error rate across three pairwise comparisons. Thus, for the purposes of the sample size calculations, we conservatively set the alpha at 0.017. We assumed that 34% of patients in the control arm will have completed their FIT, based on the National screening data [20]. Sixty-three practices with an average of 260 of eligible patients per practice (standard deviation = 197; range 51 to 753; coefficient of variation = 0.76) [15] will be sufficient to detect a difference of 10% absolute increase in participation in the NBCSP within 6 months from when the NBCSP kit is due between each intervention arm (SMS only and SMS bundle) and control arm (44% vs 34%), respectively; and to detect a smaller difference of 7.5% (44% vs 51.5%) between the SMS only and SMS bundle intervention arms. The total of 63 practices allows for an additional practice per arm for potential loss of practices due to closures or merges. A national 10% increase in screening participation would prevent 27,000 bowel cancers and 16,800 deaths and is associated with an additional $200 million in costs over current screening levels over the next 20 years [4]. We anticipate that adding the weblinks to motivational and instructional videos in the SMS would have a smaller additional effect on increasing screening participation compared to a SMS only.

#### Recruitment {15}

General practices will be identified for recruitment in the following ways: through the Department of General Practice and Primary Care at the University of Melbourne primary care practice-based research and education network, which includes general practices in Victoria and a smaller number in Queensland who are engaged with any research and/or teaching with the University of Melbourne; through the Queensland Cancer Council database of general practices who have expressed an interest in being involved in research; through the research team’s professional networks; snowballing based on advice from other practices; and cold-calling practices identified through web-based searches.

The recruitment will involve initially contacting the general practices by telephone to introduce the project and to organise a face-to-face meeting to explain the trial in more detail. If eligible (see above) and interested, the project officers will arrange to meet with the general practice staff—either face-to-face or on Zoom—to double-check the practice meets the eligibility criteria and explain the trial requirements including details about the intervention. The project officers will ensure all staff know about the trial before it starts and set up a process for staff to contact them if they have questions or to let them know if there are any staff changes during the trial. Two senior practice staff will then provide consent on behalf of the general practice.

Patients will not be individually recruited as the research team will only have access to de-identified data from the general practice that has been collected from the NCSR.

## Assignment of interventions: allocation

### Sequence generation {16a}

The unit of randomisation will be the general practice (cluster). Once all general practices have been consented and eligible participants have been identified within practices, the eligible patient lists will be sent to the NCSR to be enriched. Once the data have been sent back to the practices, the general practices will be randomly allocated with a 1:1:1 ratio to either the control or one of the two intervention arms. Randomisation will be stratified by geographical location (metropolitan/larger regional and rural/smaller regional) and state (Queensland and Victoria), and each stratum will have a computer-generated random allocation sequence with random permuted block sizes.

General practice location will be stratified as either metropolitan/larger regional if located in MMM 1–3 and rural/smaller regional if located in MMM 4–6 [17]. If two practices that share EHR are located across the two geographic locations (MMM1-3 and MMM4-6), they will be allocated to the MMM category where the main practice is located for randomisation.

### Concealment mechanism {16b}

To ensure allocation concealment the permuted block sizes will not be disclosed until all practices have been recruited and randomly allocated to the trial arms and patient data has been extracted from the EHR and linked to the NCSR data. The statistician (PC) randomising the general practices will be blinded to the identity of the participating general practices by using unique codes for each practice and will not be involved in the trial recruitment and data collection. Uninformative codes 1, 2 or 3 will be used for the trial arm allocation. Prior to random allocation, the project officers will randomly assign the uninformative codes to each of the trial arms and keep it securely stored and not disclose the key to the statisticians or the Steering committee group.

### Implementation {16c}

Following general practices' consent, and after patient data extraction from the EHR and linkage with NCSR records, the statistician (PC) will randomly allocate the general practice using the random allocation schedule and inform the project officers of the randomisation status of each general practice using the uninformative codes. Using the key for the uninformative codes, the project officers will inform the practice manager of each general practice their allocated study arm allocation both verbally and in writing. The project officers will keep a record of the practice’s unique identifier code, practice name and allocated trial arm status, which will be securely stored and only accessible by the project officers.

## Assignment of interventions: blinding

### Who will be blinded {17a}

The statisticians and the SMARTERscreen steering group members not involved in the delivery of the intervention will be masked to the general practices allocated trial arm until after the analysis of the primary outcome. General practice staff will not be blinded as to the allocation of the randomisation as this will not be possible.

### Procedure for unblinding if needed {17b}

The SMARTERscreen steering group will be unblinded as to the trial arm status code only after all the primary outcome data have been collected and analysed.

## Data collection and management

### Plans for assessment and collection of outcomes {18a}

**Fig. 4:**
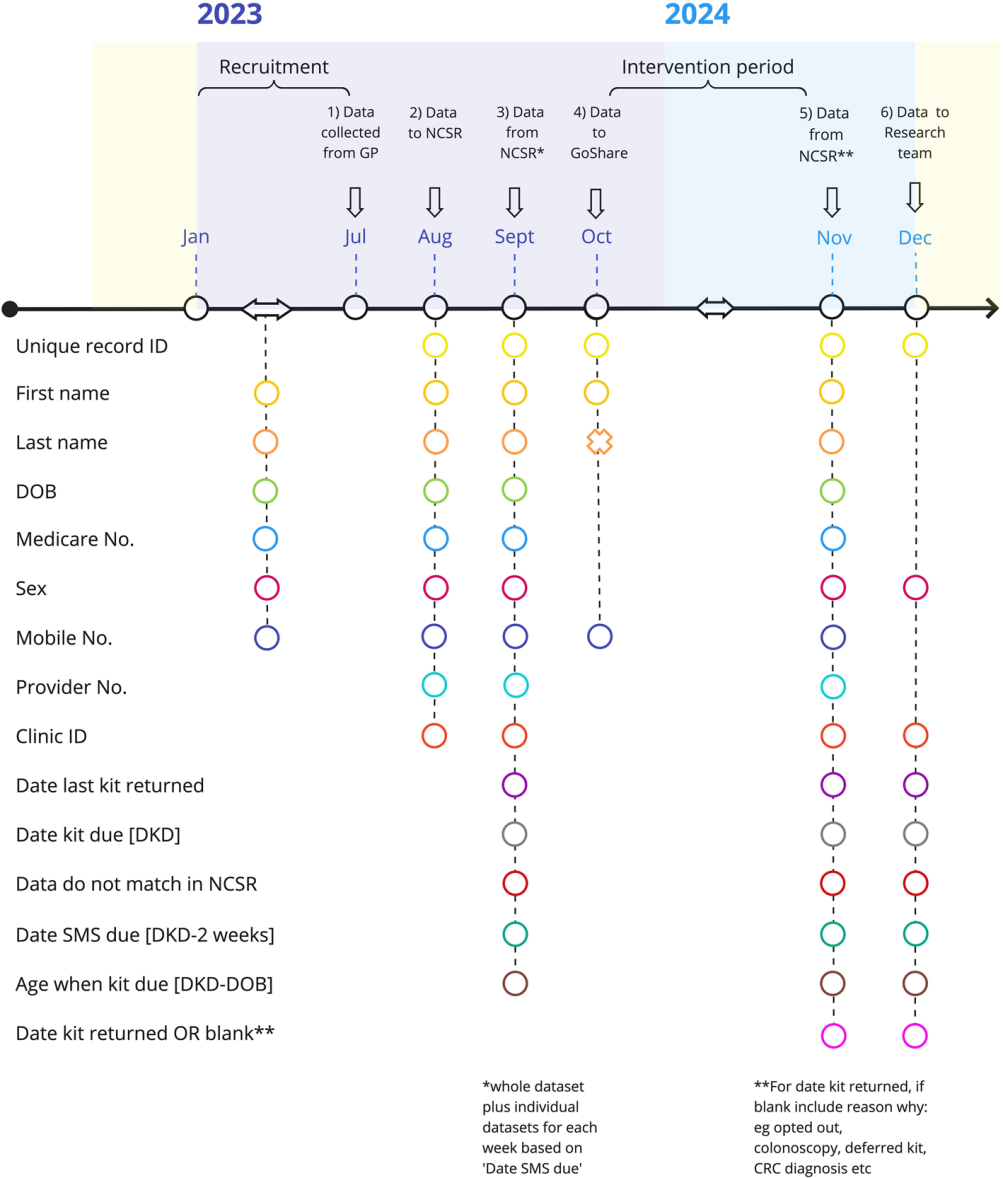
Data collection, dataset names, and timepoints (EHR, electronic health record; GP, general practice; NCSR, National Cancer Screening Register; GoShare, the SMS provider) (Proposed timeline, subject to change.)

We have developed a novel method for collecting the outcome data from the NCSR. Lists of eligible patients will be collected from general practice EHR, the NCSR will then add the dates for when each patient’s SMS will be due according to their records, and then at the end of the intervention period, the NCSR will provide the date that each patient’s kit was returned, if returned. The NCSR will send the dataset back to the general practice and a second dataset with all identifying data removed will be securely provided to the research team for analysis (Fig. 4). Depending on the NCSR capacity, to reduce the workload for the general practice and minimise risk for data errors, the de-identified dataset for analysis may be generated by NCSR and securely provided to the investigators for analysis.

The data collection at the general practice will be done within the practice by the practice manager under the guidance of the project officer and with clear instructions and technical support where necessary from the NCSR. The trial will fund a staff member at the NCSR to add the required data to the datasets at the beginning and end of the trial.

The data collection method will be tested in one practice prior to implementing the process.

#### The method (Fig. 4)

##### Step 1

The eligible patient list will be collected from each general practice EHR using a bespoke Structured Query Language (SQL) query. The list will be saved as a comma-separated values (.csv) file securely on the practice computer, with a name specific to the study identifier of the practice (practice ID) and the date of extraction (Dataset 1).

##### Step 2

The practice manager will add three columns of data including (1) a column with a Provider number for the principal GP for that practice, (2) a column with a unique trial ID code for each patient (e.g. GP0010001), and (3) a column with a unique practice ID code for each practice (e.g. GP001) (Dataset 2).

##### Step 3

This dataset will be saved as a.csv file and uploaded to the NCSR using a secure file transfer protocol (SFTP).

##### Step 4

Once the NCSR have the.csv file (Dataset 2), they will match the patients in the NCSR by date of birth, Medicare number, and name. The.csv file will be enriched with five additional columns of data for each person: one for the date they returned their last NBCSP kit (or blank if they have not returned one before) [‘Date_kit_returned’], one with the date their next NBCSP kit is due [‘Date_Kit_Due’], one if the NCSR cannot match patients’ identifying data with the register’s records [‘1’ if records match with NCSR database, ‘0’ if data in the.csv file does not match the register], one calculating the date the SMS will be sent [‘Date SMS is due’ which will be calculated as the (‘Date the NBCSP kit is due’ − 14 days)], and one calculating the patient’s age at the time when their kit is due [‘Age in months when kit is due’, which will be calculated as (= ‘Date the NBCSP kit is due’ − ‘Date of Birth’ in years, multiplied by 12)].

The NCSR will then save a.csv file (i.e. NCSR dataset) for every practice (Dataset 3).

##### Step 5

The intervention period will be for 6 months (26 weeks). The staff (funded by the research team) in the NCSR will then generate datasets for each week of the intervention period that include all patients who are due a SMS that week based on ‘Date SMS is due’ (Dataset 4). This date will be calculated so that the SMS will be sent on a Sunday for kits due 3 days on either side of that date. Each dataset will only include the Unique Record ID, Patient’s first name, and mobile number. The name of the file will identify the GP practice and the date when the SMS are due to be sent. All 26 NCSR datasets created for each general practice will be saved as separate encrypted.csv files and sent using a secure file transfer portal to the general practice.

The general practices in the intervention arms will be instructed to transfer the NCSR.csv datasets that have been separated into 26 weekly files. These will then be uploaded to the GoShare platform and scheduled for sending the SMS/SMS plus bundle on the Sunday they are due (approximately 2 weeks prior to the kit being sent). The control arm practices will not be provided access to the NCSR datasets until the end of the trial.

##### Step 6

At the end of the intervention period:

Using the Dataset 3 created in Step 5, the NCSR will add an additional variable ‘Date the kit was returned’ for each person. If there is no date for a returned kit, then a reason as to why the person was not sent the kit will be added in a separate field—this will happen if there has been a concurrent event (e.g. patient had a colonoscopy, opted out or deferred their screening, was diagnosed with colorectal cancer, or had a FIT test from elsewhere recorded), or the field will be left blank with the assumption that the person did not return the kit within 6 months of the due date of the NBCSP kit. The NCSR dataset (Dataset 5) with the added kit return dates will be stored on the NCSR secure server as a.csv dataset and downloaded by secure file transfer by the practice when needed.

##### Step 7

Dataset 6 will be created using Dataset 5, where individual identifying information (such as name, address, and mobile number) will be removed, and provided to the research team for analysis. The dataset may be securely transferred to the research team via the GP Practice (once the records have been de-identified) or directly from NCSR.

##### Step 8

Data for the measures for adherence to Interventions 1 and 2 (such as the participants who received the SMS opened and/or watched the web-based content) will be downloaded from GoShare platform and merged with Dataset 6 using the unique record ID code created in Step 2. This will be done by the project officers and Healthily.

### Plans to promote participant retention and complete follow-up {18b}

Training, including a comprehensive training manual, and ongoing support will be provided by the project officers for practice staff involved, including informing any new clinical or administrative staff who join the practice during the trial period, about the trial. The practice champion will have contact details for the project officer for their state, and contact details for the ethics committee and senior researchers. If there are any deviations from the trial or problems encountered during the study, the project officers will record them and inform the SMARTERscreen steering group.

### Data management {19}

Data management will be overseen by the project officers and under the supervision of the SMARTERscreen steering group, and statistician in accordance with the statistical analysis plan (SAP). The project officers will be responsible for training and supervising the general practice staff to extract the eligible patient list from the EHR, save it securely, name it according to the naming protocol, upload it to the NCSR, download the revised list from the NCSR, and then upload the de-identified lists with the results to the research staff (Fig. 4). The NCSR staff member will be supervised and overseen by the SMARTERscreen steering group to manage the data at the NCSR including the secure transfer to and from the general practices.

### Confidentiality {27}

All patient data will remain confidential and no identifiable patient information will be included in the final data set that is used for the trial analysis. The only people who will have access to identifiable data will be the general practice staff who already have permission to access these data, and the NCSR who also have permission to access these data. Project officers responsible for assisting and training general practice staff to collect and upload/download patient lists to the NCSR will sign confidentiality agreements between each practice and themselves and be bound by the University of Melbourne Human Research Ethics Committee requirements. Only de-identified data will be provided to the research team at the end of the data collection period with unique identifiers provided for trial participants (Fig. 4).

All general practice consent forms will be scanned and stored in a secure password-protected folder on a secure server at the University of Melbourne and only accessible to the project officer and senior researchers working on the trial. These servers are protected by a VPN and Okta verification. Any paper information will remain strictly confidential and stored in secured locked cabinets in a secure office within the Primary Care Cancer Research Group, Department of General Practice and Primary Care at the University of Melbourne and only accessible to selected researchers working on the trial (TJ, AW, SF, LB, JM). All data will be destroyed 15 years after publication according to the University of Melbourne Office of Research Ethics and Integrity Ethics Committee (OREI).

### Plans for collection, laboratory evaluation, and storage of biological specimens for genetic or molecular analysis in this trial/future use {33}

Not applicable. No biological specimens were collected.

## Statistical methods

### Statistical methods for primary and secondary outcomes {20a}

We will develop a detailed statistical analysis plan (SAP) which will be made available on the trial registry prior to conducting the primary statistical analysis. Stata 17 [21] will be used for all analyses.

Descriptive statistics will be used to compare the baseline characteristics of general practices, GPs, and patients between the three arms. Primary analysis will be intention to treat (ITT) where all general practices and their patients who receive NBCSP kit during the intervention period as determined at the beginning of the trial period will be analysed in the arm that they were allocated to, regardless of whether they received all or part of the intended intervention. For the primary outcome, logistic regression and generalised linear model with an identity link function and binomial family (when appropriate) will be used to estimate the odds ratio (relative measure) and difference in proportions (absolute measure) of each intervention compared to the control arm, and Intervention 1 compared to Intervention 2. Both regression models will use generalised estimating equations with robust standard errors to allow for clustering by general practice and will adjust for geographical remoteness (metropolitan/larger regional and rural/smaller regional) and state (Queensland and Victoria). Estimates of the intervention effect will be reported as both differences in the proportion (absolute measures) and odds ratio (relative measure) for each pair-wise comparison (control vs SMS only, control vs SMS bundle, SMS only vs SMS bundle) with respective 95% confidence interval and an overall *p*-value value testing the global null hypothesis of no difference in the proportion of eligible patients who return their FIT kit within 6 months of the due date across the three arms. No adjustments will be made for the multiple comparisons [22].

We will also estimate the intra-general practice correlation coefficient for the primary outcome, which quantifies the proportion of the true total variation in the outcome attributable to between-cluster variation, and this will be estimated and reported with 95% confidence intervals.

### Interim analyses {21b}

No interim analysis is planned.

### Methods for additional analyses (e.g. subgroup analyses) {20b}

We will conduct a sensitivity analysis for the primary outcome; we will adjust for pre-specified baseline covariates, such as sex and age of the patient, and whether they have ever or never screened previously (according to the NCSR data). To address aim 2, we will conduct a sub-group analysis separately for each patient characteristic: age, sex, previous screening, and location of practice. For sub-group analysis we will include an interaction between patient characteristic and trial arm in the regression model described above for the primary analysis.

### Methods in analysis to handle protocol non-adherence and any statistical methods to handle missing data {20c}

A blinded review of the data will inform the approach for handling of missing outcomes. Supplementary analyses, including sub-group and adherence-adjusted analyses, handling of missing data, and sensitivity analysis to assess model assumptions including the robustness of the missing data assumption, will be detailed in the SAP.

#### Evaluation of adherence to intervention

Descriptive statistics will be used to evaluate adherence to the two interventions, overall and by general practice location, participant sex, and age. Counts and proportions will be used for the binary measures by each Intervention. For Intervention 2, of individuals who opened the bundle at least once, the number of times a weblink in the SMS bundle is clicked on, the number of times each of the two videos are viewed, and webpage viewed will be presented as total counts, and rates per individual, respectively.

#### Economic evaluation

Led by JBL, the economic evaluation will be conducted using an existing calibrated and validated microsimulation platform, Policy1-Bowel, developed by the Daffodil Centre [7, 23] The model has been used to evaluate the health benefits, burden and harms, and cost-effectiveness of different bowel cancer screening approaches to inform the bowel cancer screening policy in Australia [7, 23]. In brief, the model simulates the life histories of bowel lesion(s) (conventional adenoma and sessile serrated lesion) and cancer development, bowel cancer survival, and bowel cancer screening in individuals in Australian population. Each simulated individual could develop up to ten adenomas and ten serrated lesions simultaneously. The simulated individuals who have advanced adenoma(s) (i.e. a conventional adenoma that is large, with high-grade dysplasia, or with villous histology) and/or sessile serrated lesion(s) have an annual risk of developing into a preclinical cancer. Over time, a preclinical cancer can progress to a more advanced stage or become clinically diagnosed due to symptoms or bowel cancer screening. Patients diagnosed with bowel cancer have a risk of dying, which varies by cancer stage at diagnosis and time since cancer diagnosis. In the model, patients who survive for 5 years after cancer diagnosis are assumed to no longer be affected by bowel cancer and have no additional risk of dying from bowel cancer compared with the average population with no bowel cancer.

For this economic evaluation, the NBCSP participation rates for each intervention arm in the trial and the costs associated with sending a GP practice-endorsed SMS with/without additional motivational material will be incorporated into the Policy1-Bowel model. Cost-effectiveness and the difference in the 5-, 10- and 20-year bowel cancer incidence and mortality outcomes among participants of the two SMS intervention arms versus the control arm will be estimated. Furthermore, the model will also be used to estimate the budget impact on the health care cost and the 5-, 10-, and 20-year cancer incidence and mortality reduction in the Australian population if the SMS intervention was adopted and implemented nationwide compared with the current practice.

### Plans to give access to the full protocol, participant-level data, and statistical code {31c}

To assist with reproducible research, the full protocol, non-identifiable participant-level data, and statistical code will be made available to external researchers upon reasonable request. The steering committee will manage external requests for these materials.

## Oversight and monitoring

### Composition of the coordinating centre and trial steering committee {5d}

The investigator team includes JM, JE, PC, BG, CW, JT, SC, JH, TC, FM, JBL, KM, CN, ID, MC, NL, LI, TJ, SD, KB, GA, JJ, and MJ and the trial steering group includes JM, JE, PC, BG, CW, JT, and MJ. The steering committee is responsible for designing the trial protocol, data collection plan, statistical analysis plan, trial conduct, ethical conduct, budget, contractual obligations, and research staff management.

### Composition of the data monitoring committee, its role and reporting structure {21a}

JT, PC, JM, AW, TJ, JE, SF, and MJ will report to the investigators as to the data collection and analysis plan.

### Adverse event reporting and harms {22}

Any adverse events and other unintended effects that may arise from the trial intervention will be reported to the University of Melbourne Office of Research Ethics and Integrity Ethics Committee (OREI).

### Frequency and plans for auditing trial conduct {23}

Progress reports will be submitted annually to the University of Melbourne Office of Research Ethics and Integrity (OREI) and regularly to the Australian and New Zealand Clinical Trials Registry (ANZCTR). This will be completed by the Project Lead JM. Progress will be reported to the investigators with quarterly meetings.

### Plans for communicating important protocol amendments to relevant parties (e.g. trial participants, ethical committees) {25}

Any amendments to the protocol will be discussed in the weekly meetings with the SMARTERscreen steering group and project officers (JM, JE, PC, BG, JT, LB, SF, and MJ) and protocol amendments will be communicated to the investigators by email and at quarterly meetings. The project officers will communicate with the rest of the steering committee to ensure they are all involved in the decision-making. They will also inform the ethics committee (OREI) and the trial register (ANZCTR) with modifications to the protocol or progress of the trial as necessary.

## Dissemination plans {31a}

SPIRIT guidance: Plans for investigators and sponsor to communicate trial results to participants, healthcare professionals, the public, and other relevant groups (e.g. via publication, reporting in results databases, or other data sharing arrangements), including any publication restrictions.

## Discussion

This protocol describes the trial design informed by the SMARTscreen trial which demonstrated that using an SMS with a combination of additional features including endorsement by a primary care clinician, a motivational video, instructions for how to do the NBCSP kit, and links to extra information was efficacious for increasing bowel cancer screening [15]. This trial—‘SMARTERscreen’—will address the limitations we found in SMARTscreen which included a potential lack of generalisability as we only included regional practices from one state in Australia, the use of incomplete data as the data used to calculate the results were from general practice electronic health records, and we only had aggregated data at the practice level.

Increasing participation in the Australian NBCSP has the potential to reduce bowel cancer incidence and reduce associated health costs over 20 years [7] and bring the Australian screening programme in line with international bowel cancer screening programmes which have much higher participation rates of 60–70% [24]. This is one of the health priorities of the Australian Government.

## Conclusion

This trial will build on previous research conducted by this research group and has the potential to demonstrate the effectiveness of a simple technological intervention to improve screening uptake which is scalable and sustainable.

## Trial status

The SMARTERscreen trial has approval from the Human Research Ethics Committee at the University of Melbourne and started recruitment on 12 February 2023. Protocol is dated 7 June 2023; version 1.0.

All practices have been recruited (21 July 2023) and we anticipate data extraction from the NCSR will begin in September 2023. The intervention period will begin once recruitment and baseline data have been collected. The intervention will begin in early 2024 once individuals’ eligibility is determined and randomisation is implemented.

## Data Availability

The trial data set will be available to the trial coordinator and the statistician. These data including the statistical code will not be available for public access.

## Abbreviations

ANZCTR: Australia New Zealand Clinical Trial Registry
CRC: Colorectal Cancer
EHR: Electronic Health Record (i.e. general practice patient record)
FIT: Immunochemical Faecal Occult Blood Test
GP: General Practitioner
HPP: Health Provider Portal
ITT: Intention To Treat analysis
NBCSP: National Bowel Cancer Screening Program
NCSR: National Cancer Screening Register
PRODA: Provider Digital Access
RCT: Randomised Controlled Trial
SAP: Statistical Analysis Plan
SMS: Short Messaging Service
